# Crosstalk between RNA-Binding Proteins and Immune Microenvironment Revealed Two RBP Regulatory Patterns with Distinct Immunophenotypes in Periodontitis

**DOI:** 10.1155/2021/5588429

**Published:** 2021-07-05

**Authors:** Lu Xing, Guanqun Meng, Tian Chen, Xiaoqi Zhang, Ding Bai, Hui Xu

**Affiliations:** ^1^State Key Laboratory of Oral Diseases & National Clinical Research Center for Oral Diseases, Sichuan University, Chengdu, China; ^2^Department of Population and Quantitative Health Sciences, School of Medicine, Case Western Reserve University, Cleveland, OH 44106, USA; ^3^State Key Laboratory of Oral Diseases & National Clinical Research Center for Oral Diseases; Department of Orthodontics, West China Hospital of Stomatology, Sichuan University, Chengdu, China

## Abstract

Periodontitis is an inflammatory disease whose pathogenesis is closely related with immunology. RNA-binding proteins (RBPs) were found to play crucial roles in immunity. Therefore, we aimed to investigate the potential impact of RBPs in the immune microenvironment in periodontitis. The differential expressions of RBPs in periodontitis and healthy samples were determined and were used to construct an RBP-based classifier for periodontitis using logistic regression. The correlations between RBPs and immune characteristics were investigated by the Spearman correlation. Unsupervised clustering was conducted to identify the RBP regulatory patterns. RBP-related genes were identified by WGCNA, while biological distinctions were revealed by GSVA and GO. 24 dysregulated RBPs were identified, from which a 12-RBP classifier was established to distinguish periodontitis with AUC of 0.942. Close protein-protein interactions and expression correlations were observed especially between SPATS2 and ISG20. ISG20 and ESRP1 were found to be highly correlated with immunocyte infiltration, immune signaling activation, and HLA expressions in periodontitis. Two distinct RBP regulatory patterns were identified with different immune and other biological characteristics in periodontitis. Our findings indicate a significant impact of RBPs in shaping the immune microenvironment in periodontitis, which might bring new insights into the understanding of immune mechanisms in the pathogenesis of periodontitis.

## 1. Introduction

Periodontitis is an inflammatory disease initiated by bacteria infection. It detrimentally affects periodontal supporting tissues, causing symptoms such as swelling of gingiva, periodontal pyorrhea, and tooth loosening [1]. It is reported that severe periodontitis is the sixth most prevalent health condition, affecting 10.8% of the population around the globe [2]. It brings about severe health and economic burdens, as prosthodontic cost for tooth loss caused by periodontitis is usually not a small budget [2, 3]. Over the years, periodontitis have been implicated as an etiological factor in systemic diseases such as diabetes, rheumatoid arthritis, and cardiovascular diseases [4]. Unfortunately, treatment for periodontitis has thus far failed to reverse the tissue damage, which means that actually there is currently no cure for periodontitis [5].

The initiation of periodontitis can be recognized to be a cascade of immune/inflammatory responses that was triggered by periodontal pathogens. The degree of periodontal damage relies heavily on the host response, particularly on the inflammatory process and the activation patterns of immune response pathways during periodontitis [6]. Failure to resolve inflammation and attempt to restore tissue homeostasis cause neutrophil-mediated destruction in both the alveolar bone and extracellular matrix [7]. The inflammatory reaction, rather than the pathogens, causes irreversible damage in the periodontal tissue. Thus, a promising therapy for periodontitis is to resolve inflammation and return tissue to homeostasis. Elucidation of the mechanisms of immune regulations in periodontitis is crucial to the development of novel treatment strategies.

RNA-binding proteins (RBPs) are a large group of proteins that bind to RNA either directly or as a part of a macromolecular complex. As a critical part of the posttranscriptional gene regulator, RBPs facilitate the maturation, stability, transportation, and degradation of cellular RNAs [8]. RBPs play pivotal roles in cell development and stress response, and its dysregulation could certainly cause diseases [9]. Various types of RBPs have been identified to be implicated in the maintenance of immune homeostasis [10]. For instance, conditional deletion of Elavl1 caused impediment to immune cell development [11, 12]. hnRNPC was involved in follicular B cell maintenance [13]. Based on the immunoregulatory role of RBPs and immunomicrobial pathogenesis of periodontitis, it is plausible to deduce that RBPs might play a crucial part in periodontitis. The RBP HuR was reported to modulate inflammatory responses in periodontitis by regulating IL-6 [14]. However, evidence on the regulatory role of RBPs in periodontitis is quite rare. Systematic analyses exploring the functions of RBPs and their roles in shaping the immune microenvironment in periodontitis are warranted.

Considering the unveiled role of RBPs in periodontitis and involvement of RBPs in immunoregulation, this study is aimed at portraying the overall landscape of RBPs in periodontitis and uncovering its implications with the immune microenvironment of periodontitis. The findings are expected to reveal the pathogenesis of periodontitis in the perspective of RBP-mediated immunoregulatory mechanism.

## 2. Results

### 2.1. Expression Landscape of RBPs in Periodontitis

**Figure 1:**
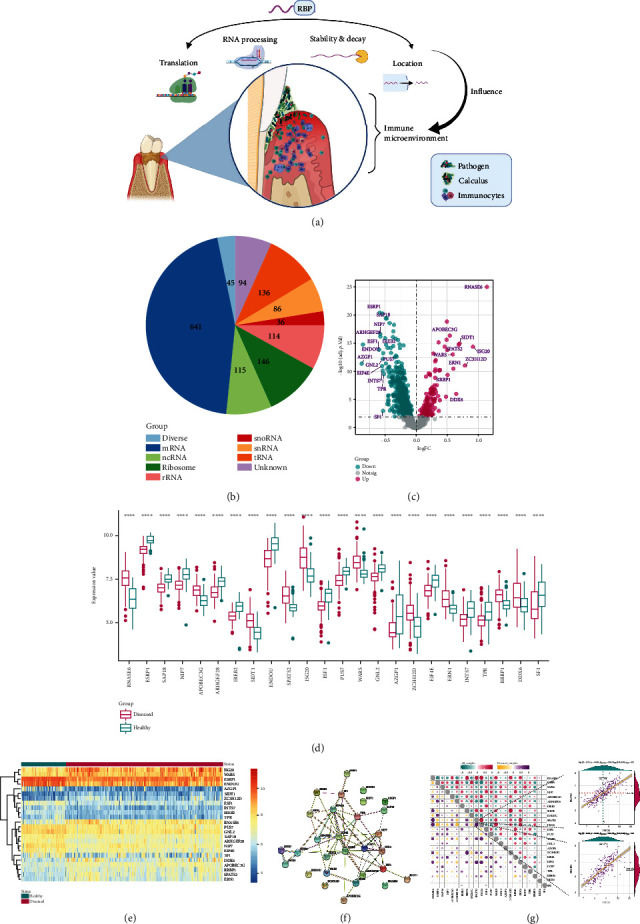
Expression landscape of RNA binding proteins (RBPs) in periodontitis. (a) The overview of RBPs' regulatory role in periodontitis. (b) A summary of the types of RNA which the RBPs were binding to. (c) The volcano plot demonstrated the differentially expressed RBPs in periodontitis and periodontally healthy samples. RBPs with adjust *p* value < 0.01 and ∣logFC | >0.5 were considered to be significantly dysregulated, and their gene names were marked. The box plot (d) and heatmap (e) demonstrated the expression status of the 24 dysregulated RBPs between periodontally healthy and periodontitis samples. (f) The protein-protein interaction network of the 24 dysregulated RBPs. (g) The correlation among the 24 significantly dysregulated RBPs in periodontitis samples and whole samples. The most correlated RBPs in all samples and periodontitis samples were demonstrated in the dot plot.

The overall regulatory mechanisms of RBPs in the immune microenvironment in periodontitis were presented in Figure 1(a). The RBP gene list was obtained from a previous research [15]. The types of RNA which the RBPs were binding to were concluded in the pie chart (Figure 1(b)). Differential analysis revealed that 24 RBPs were significantly dysregulated between periodontally healthy and periodontitis samples (adjust *p* value < 0.01 and ∣logFC | >0.5, Figure 1(c), Table S1). Box plot and heatmap demonstrated the expression status of the 24 dysregulated RBPs (Figures 1(d) and 1(e)). To figure out the interaction relationship of these dysregulated RBPs, a protein-protein interaction network was constructed (Figure 1(f)), and their expression correlation relationship was calculated by correlation analysis. It was found that the most positively correlated pair is ZC3H12D-SIDT1 while the most negatively correlated pair is ISG20-ESRP1 (Figure 1(g)).

### 2.2. Differential Expression Patterns of RBPs between Periodontitis and Periodontally Healthy Samples

**Figure 2:**
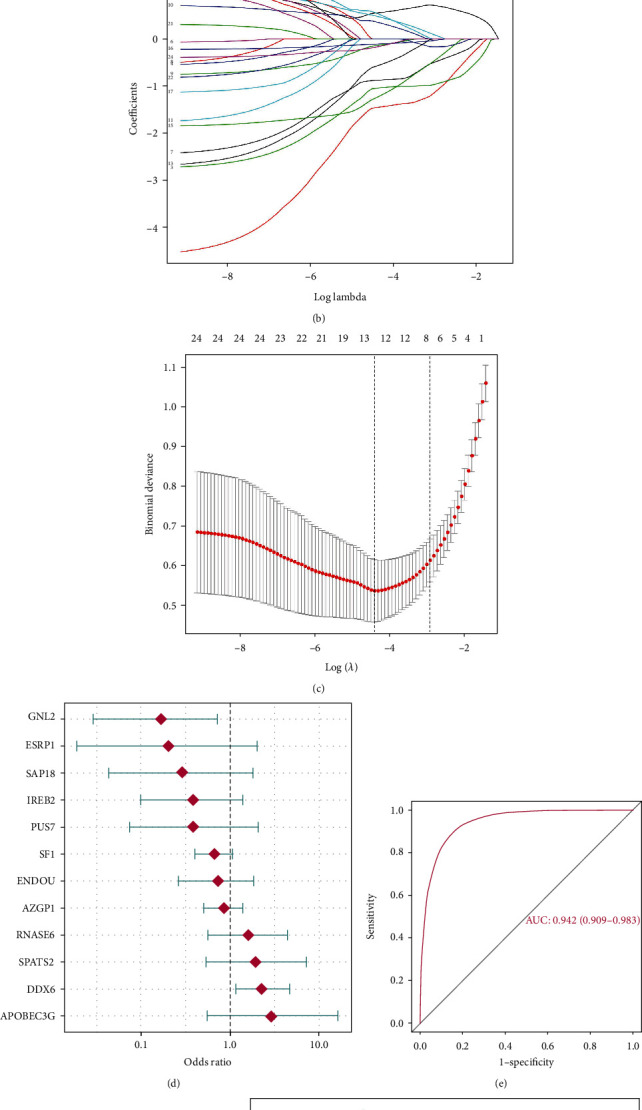
RBPs can well distinguish periodontally healthy and periodontitis samples. (a) Univariate logistic regression analysis was performed on the 24 dysregulated RBPs. (b) Least absolute shrinkage and selection operator (LASSO) regression coefficients of the 24 dysregulated RBPs. (c) Tenfold crossvalidation for tuning parameter selection in LASSO regression. The partial likelihood deviance is plotted against log (*λ*), where *λ* is the tuning parameter. Partial likelihood deviance values are shown, with error bars representing SE. The dotted vertical lines are drawn at the optimal values by minimum criteria and 1-SE criteria. (d) Multivariate logistic regression analysis was performed to establish a 12-RBP classifier. (e) Receiver operating characteristic (ROC) analysis evaluated the discrimination ability of the 12-RBP classifier. (f) Risk score distribution of periodontitis and periodontally healthy samples. (g) PCA analysis of the periodontitis and periodontally healthy samples based on the expression of the 12 RBPs.

To further validate the distinction of RBPs expressions between periodontally healthy and periodontitis samples, logistic regression was conducted. Univariate logistic regression analysis was performed on the 24 significantly dysregulated RBPs, and their odds ratio were presented on the forest plot with the 95% confidence interval (Figure 2(a), Table S2). It was found that they were all significantly related with periodontitis (adjust *p* value < 0.05). To make dimension reduction and remove unimportant features, we performed least absolute shrinkage and selection operator (LASSO) regression for feature selection and reduce overfitting of the model, and 12 RBPs were identified with the lambda of 0.0146 (Figures 2(b) and 2(c)). Multivariate logistic regression analysis was performed on the 12 RBPs to construct a 12-RBP classifier for periodontitis (Figure 2(d), Table S3), with the risk score calculated for each of the samples (Figure 2(f)). Receiver operating Characteristic (ROC) analysis revealed that the classifier had excellent discriminative ability with the area under the curve (AUC) of 0.942 (Figure 2(e)). Periodontitis samples had much higher risk scores compared with periodontally healthy ones (Figure 2(f)). PCA analysis based on the 12 RBPs suggests that periodontitis and periodontally healthy samples had distinct expression patterns of the 12 RBPs (Figure 2(g)).

### 2.3. Immune Microenvironment Characteristics in Periodontitis and Their Correlations with RBPs

The immune microenvironment of periodontitis was explored in 241 periodontitis and 69 periodontally healthy samples. In brief, relative enrichment score of immunocytes, relative activity of immune-related pathways, and expression of HLA were calculated, and their correlations with RBPs were investigated.

**Figure 3:**
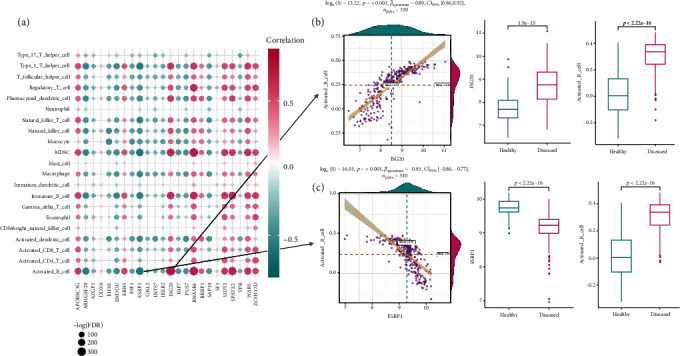
Immunocyte infiltration status in periodontitis and their correlation with RBPs. (a) Correlation analysis between relative enrichment score of immunocytes and RBP expression levels. (b) Dot plot and box plot reveal the relationship between the most positively correlated immunocyte-RBP pair, activated_B_cell, and ISG20, with a higher enrichment score of activated_B_cell and higher expression of ISG20 in periodontitis samples. (c) Dot plot and box plot reveal the relationship between the most negatively correlated immunocyte-RBP pair, activated_B_cell, and ESRP1. A higher enrichment score of activated_B_cell and lower expression of ESRP1 were observed in periodontitis samples.

The majority of the types of immunocytes showed significantly increased infiltration in periodontitis samples compared with periodontally healthy ones (*p* < 0.05) (Figure S1A, Table S4). The most positively correlated immunocyte-RBP pair is activated B cell and ISG20, both significantly upregulated in periodontitis. The most negatively correlated immunocyte-RBP pair is activated B cell and ESRP1, with significant downregulation of ESRP1 in periodontitis (Figures 3(a)–3(c), Table S5).

**Figure 4:**
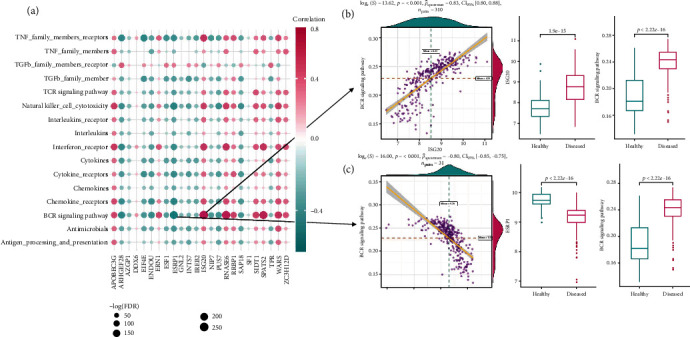
Immune-related pathways in periodontitis and their correlation with RBPs. (a) Correlation analysis between activities of immune pathways and RBPs expression levels. (b) Dot plot and box plot reveal the relationship between the most positively correlated immune pathway-RBP pair, BCR signaling pathway, and ISG20. A higher activity of BCR signaling pathway and higher expression of ISG20 were observed in periodontitis samples. (c) Dot plot and box plot reveal the relationship between the most negatively correlated immune pathway-RBP pair, BCR signaling pathway, and ESRP1. A higher activity of BCR signaling pathway and lower expression of ESRP1 were observed in periodontitis samples.

**Figure 5:**
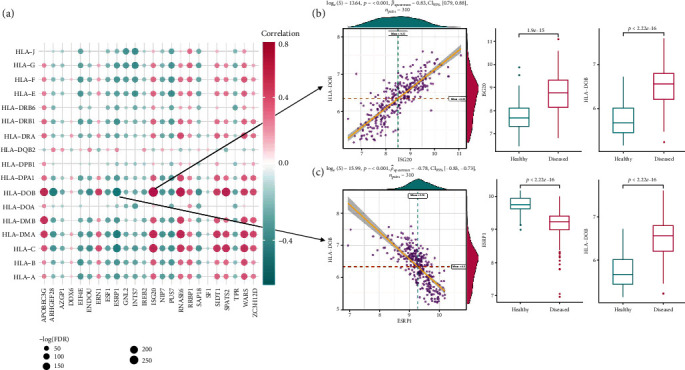
HLA expression status in periodontitis and their correlation with RBPs. (a) Correlation analysis between HLA and RBP expression levels. (b) Dot plot and box plot reveal the relationship between the most positively correlated HLA-RBP pair, HLA-DOB, and ISG20. Higher expressions of HLA-DOB and ISG20 were observed in periodontitis samples. (c) Dot plot and box plot reveal the relationship between the most negatively correlated HLA-RBP pair, HLA-DOB, and ESRP1. Higher expression of HLA-DOB and lower expression of ESRP1 were observed in periodontitis samples.

Similarly, as for immune-related pathways, almost all are significantly activated in periodontitis, except for TGFb family member receptor which had a significantly lower activity (Figure S1B, Table S6). Correlation analysis demonstrated that the most positively correlated immune pathway-RBP pair is BCR signaling pathway (B cell receptor signaling pathway) and ISG20, with higher activities of both in periodontitis. The most negatively correlated pair is BCR signaling pathway and ESRP1, with a higher activity of BCR signaling pathway and a lower expression of ESRP1 in periodontitis (Figures 4(a)–4(c), Table S7). Similar results were also found in HLA expression. Almost all the HLA genes were significantly upregulated in periodontitis, except for HLA-DQB2 which showed a significantly lower expression (Figure S1C, Table S8). Correlation analysis revealed HLA-DOB and ISG20 as the most positively correlated HLA-RBP pair, with higher activities of both in periodontitis. The most negatively correlated pair is HLA-DOB and ESRP1, with a higher expression of HLA-DOB and a lower expression of ESRP1 (Figures 5(a)–5(c), Table S9).

These findings demonstrated strong correlations of the RBPs ISG20 and ESRP1 with activated B cell infiltration, BCR signaling activation, and HLA-DOB expression, which were among the immune characteristics showing the most significant difference between periodontitis and periodontally healthy samples.

### 2.4. Identification of Distinct RBP Regulatory Patterns within Periodontitis Samples

**Figure 6:**
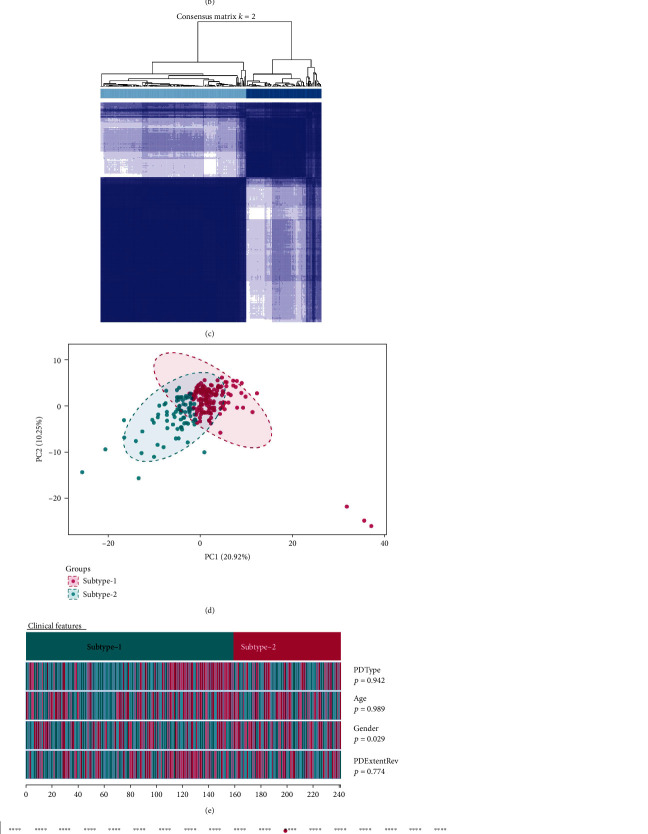
Unsupervised clustering of periodontitis samples based on the RBP expression. (a) Consensus clustering cumulative distribution function (CDF) for *k* = 2–7. (b) Relative change in area under the CDF curve for *k* = 2–7. (c) Heatmap of the matrix of cooccurrence proportions for periodontitis samples. (d) Principle component analysis (PCA) of the two RBP regulatory subtypes. (e) Clinical features of the two RBP regulatory subtypes. (f) Expression of subtype-specific RBPs. The subtype-specific RBPs were the differentially expressed RBPs between the two RBP regulatory subtypes (adjust *p* value < 0.01, ∣logFC | >0.6). (g) Heatmap of the expression of subtype-specific RBPs.

Since RBPs had been linked with periodontal immune homeostasis, we clustered the samples based on its RBP expression to see if subtypes could be observed within periodontitis samples. Unsupervised consensus clustering analysis was performed on the 241 periodontitis samples based on their RBP expressions and identified two subtypes (Figures 6(a)–6(c), Table S10). PCA analysis demonstrated that the two subtypes had distinct RBP regulatory patterns (Figure 6(d)). Furthermore, we compared the clinical characteristics and found that there was a significant difference in gender between the two subtypes (Figure 6(e)). The subtype-specific RBPs were identified, showing different expression patterns between the two subtypes (adjust *p* value < 0.01, ∣logFC | >0.6, Figures 6(f) and 6(g), Table S11).

### 2.5. Distinct Immune Characteristics Were Observed between Two RBP Regulatory Patterns

**Figure 7:**
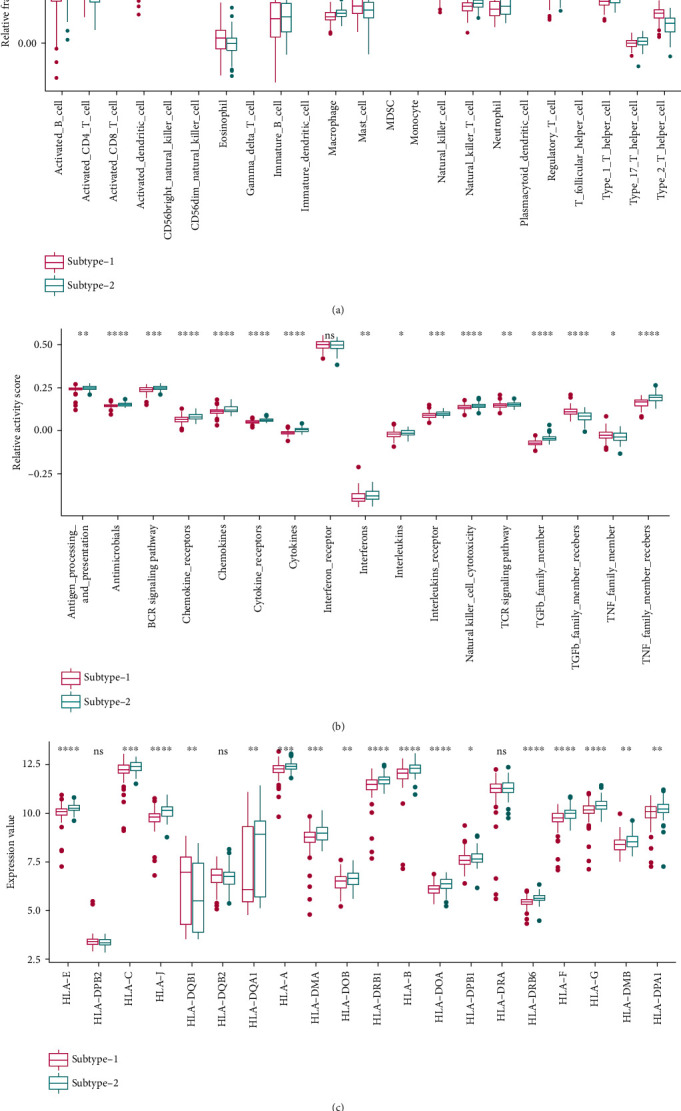
Immune characteristics in the two RBP regulatory subtypes. (a) Immunocyte infiltration status in the two RBP regulatory subtypes. (b) Activity of immune pathways in the two RBP regulatory subtypes. (c) HLA expression levels in the two RBP regulatory subtypes.

Considering the strong correlations found between RBPs and the immune microenvironment, we looked further into the subtypes to see if different RBP regulatory patterns correspond to distinct immune characteristics. Subtype-2 demonstrated more intense immune reactions, with higher relative enrichment scores of immunocytes, higher activities of immune-related pathways, and higher HLA expressions. For instance, the aforementioned activated B cell and HLA-DOB, which fell into the most correlated immunocyte-RBP and HLA-RBP pairs, respectively, were significantly upregulated in subtype-2 compared with subtype-1. In addition, the BCR signaling pathway, which belonged to the aforementioned most correlated immune pathway-RBP pair, had higher activity in subtype-2. These findings linked two RBP regulatory patterns to distinct immune characteristics in periodontitis (Figures 7(a)–7(c)).

### 2.6. Biological Distinctions between the Two RBP Regulatory Patterns

To figure out the biological reactions happening under the two RBP regulatory patterns, Gene Set Variation Analysis (GSVA) on Hallmarks and KEGG pathways was employed which revealed biological pathway differences in the two subtypes, respectively (Figure S2A and B). Then, in order to find what caused the biological differences between the two RBP regulatory patterns, we identified RBP phenotype-related genes and employed GO-BP functional enrichment analysis on them (Figure S2C). To find out if biological differences occurred specifically regarding immunity, we employed GO-BP functional enrichment analysis on RBP phenotype-related immune genes and clustered them according to the function, and those genes were mostly enriched on immune receptor related pathways such as the Fc receptor signaling pathway and immune response-regulating cell surface receptor signaling pathway (Figure S2D). Furthermore, to identify gene modules involved in the two RBP regulatory subtypes, WGCNA was employed. 22 gene modules were identified, and we performed correlation analysis of those gene modules with the two subtypes. We found that each RBP regulatory pattern had their respective matching gene modules. The modules mostly positively correlated with subtype-1 or subtype-2 were represented by blue or brown, respectively. (Figure S3A-D, Table S12).

## 3. Materials and Methods

### 3.1. Data Preprocessing

The 310 samples included in this study (69 periodontally healthy samples and 241 periodontitis samples) came from 120 patients that underwent periodontal surgery [16]. The procedure of sample procession and RNA extraction were described in the previous study [16]. The gene expression was detected by Affymetrix Human Genome U133 Plus 2.0 Array microarray according to the manufacturer's instructions [16]. The data was reserved in the GEO database under the serial number GSE16134 (https://www.ncbi.nlm.nih.gov/geo/query/acc.cgi?acc=gse16134) and obtained by the R package “GEOquery.” CEL files in the series were processed by “RMA” package in R with “justRMA” function under default parameters. Probes were annotated as gene symbols, and probes without matching gene symbols or had multiple matching gene symbols were excluded. Expressions of duplicate genes were calculated as the median value. Normalization of the gene expression was processed by “normalizeBetweenArrays” in the R package “limma.” The 1542 RBP gene list used in this study was obtained from a previous research screening for human RBPs [15] R version: 3.6.1. The overall regulatory mechanisms of RBPs in the immune microenvironment of periodontitis were presented in the graphical abstract which was created with http://biorender.com/.

### 3.2. Identification of Dysregulated RBPs and the Construction of the RBP Classifier

Dysregulated RBPs were evaluated by the “limma” package with adjust *p* value < 0.01 and ∣logFoldChange | >0.5. The protein-protein network of the dysregulated RBPs was constructed by the online database STRING (https://string-db.org/). Correlation analyses of the dysregulated RBPs as well as other correlation analysis in this study were conducted by the Spearman correlation analysis. Univariate logistic regression, LASSO regression, and multivariate logistic regression were used to establish the 12-RBP classifier and receiver operating characteristic (ROC) analyses were used to evaluate its classification ability.

### 3.3. Quantitative Evaluation of Immune Microenvironment in Periodontitis

The evaluation of the overall status of immune infiltration in periodontitis and healthy samples was conducted using the same method as we have illustrated in the previous study, and the results were consistent [17, 18]. In brief, single-sample Gene Set Enrichment Analysis (ssGSEA) was conducted to evaluate the relative enrichment score of immunocytes and the activity of immune-related pathways. The gene sets used in ssGSEA for immune-related pathway evaluation were from the online database Immport (http://www.immport.org) [19]. The comparisons of relative enrichment score of immunocytes [20], activity of immune-related pathways, and expression of HLA between periodontitis and periodontally healthy samples were conducted using Wilcox test; *p* < 0.05 was considered to be significant.

### 3.4. Identification of RBP Regulatory Patterns

Based on the expressions of the 1542 RBP genes, the periodontitis samples were clustered into two subtypes using unsupervised clustering analysis. The cluster numbers and robustness were evaluated by consensus clustering algorithm [20, 21]. The R package “ConsensuClusterPlus” was used to conduct the steps described above for 1000 iterations in order to guarantee the robustness of the clustering [22]. The comparisons of relative enrichment score of immunocytes, activity of immune-related pathways, and expression of HLA between the two subtypes were conducted using the Wilcoxon test.

### 3.5. Biological Functions of the Two RBP Regulatory Patterns

Hallmarks and KEGG pathways were used to summarize the biological functions and distinction of the two RBP regulatory patterns. Gene Set Variation Analysis was used to evaluate enrichment levels, and the R package “limma” was used to compare between the two subtypes. Pathways with *p* value < 0.01 were considered to be significant. The gene sets were from “h.all.v7.0.symbols” and “c2.cp.kegg.v7.0.symbols” which were downloaded from the MSigDB database. The GO-BP enrichment analysis of the RBP phenotype-related genes and immune genes was conducted by the “clusterProfiler” package. In addition, to identify gene modules related to RBP regulatory patterns, weighted gene coexpression network analysis (WGCNA) was employed on periodontitis samples using “WGCNA” package. Correlation analysis between gene modules and subtypes was conducted with the Pearson correlation analysis.

## 4. Discussion

Periodontitis is a complex infectious disease, and dysregulation of innate and adaptive immunity plays a key role in the etiology [23]. With more knowledge of RBP regulatory mechanisms, more evidences show that RBPs play a significant role in the initiation and regulation of immune responses [24]. Our study identified 24 significantly dysregulated RBPs to distinguish periodontitis from periodontally healthy samples, with 12 of them selected to compose a molecular classifier for periodontitis, and revealed two RBP regulatory subtypes corresponding to distinct immunophenotypes in periodontitis, with two gene modules significantly correlated with the division of the two subtypes. It is by far the first evidence on systematic evaluation of the role of RPBs in the immune microenvironment in periodontitis.

In this study, the immune microenvironment of periodontitis was found to be characterized by increased infiltration of immunocytes, higher activities of immune-related pathways, and upregulated HLA expression, among which, activated B cells, BCR signaling pathway, and HLA-DOB were ones of those showing the most significant difference from periodontally healthy samples, as well as being significantly upregulated in the subtype of periodontitis with more intense immune reactions. Interestingly, those three were also the ones having the most significant correlations with RBP expressions. Paired with the immune characteristics above, the most strongly correlated RBPs were ISG20 and ESRP1, suggesting that ISG20 and ESRP1 might have potent impact on the immune microenvironment in periodontitis.

ISG20 (interferon-stimulated exonuclease gene 20) responds to interferon and exerts its antiviral abilities by binding to single-stranded RNA and acts as exonuclease to degrade viral RNAs. It mainly targets RNA viruses including hepatitis C virus (HCV), hepatitis A virus (HAV), and yellow fever virus (YFV) [25]. Higher expression of ISG20 was associated with suppressed adaptive immune responses, increased infiltration of monocyte-derived macrophages and neutrophils, higher tumor grade, and poorer clinical outcome in glioma [26]. In chronic periodontitis, researchers detected aberrantly upregulated ISG20 genes in monocytes stimulated by LPS from *Porphyromonas gingivalis* [27]. ESRP1 encodes a mRNA splicing factor that regulates the formation of epithelial cell-specific isoforms [28]. In melanoma, patients with a lower expression of ESRP1 expressed mesenchymal markers and higher level of immune cytolytic activity and experienced better survival rate [29]. In our study, ISG20 was found to be highly positively correlated with activated B cell infiltration, BCR signaling activity, and HLA-DOB expression, while ESRP1 was highly negatively correlated with the above. In periodontitis, B cells initiate immune responses by producing antibodies against periodontal pathogens, and activated B cells could serve as antigen presenting cells towards CD4+ and CD8+ T cells [30]. In individuals more susceptible to periodontitis, B cells exhibited more autoreactive properties [31]. HLA-DOB belongs to the MHC class II beta chain which forms HLA-DO to interact with HLA-DM on B cells in the antigen presentation process [32]. The strong associations among ISG20, ESRP1 and activated B cell, BCR signaling pathway, and HLA-DOB in periodontitis were first identified in our study. Our findings might help to reveal the pathogenesis of periodontitis in the perspective of RBP-related molecular biology and to find novel immune therapeutic target for periodontitis.

Two RBP regulatory subtypes of periodontitis we have identified exhibited distinct immune profiles, with subtype-2 having increased infiltration of immunocytes, and higher activities of immune-related pathways and expressions of HLA compared with subtype-1, indicating more intense immune reactions in subtype-2. Furthermore, GSVA and functional enrichment analyses revealed other biological distinctions between the two RBP regulatory subtypes aside from the immune aspects. This RBP-based clustering method not only gave distinct division of immunophenotypes, but also sorted out different biological profiles in periodontitis. The possible associations among the RBPs, immunophenotypes, and other biological signaling are of interest for further investigations.

Since RBP regulatory subtypes have been linked with distinct periodontal immune microenvironment and biological profiles, comparing the clinical information between the subtypes is meaningful and worthwhile. However, a limitation of this study was insufficient information on clinical features (only gender, age, and PD type were recorded). Therefore, this comparison could not reflect the entire profile of clinical characteristics. We found a significant difference in gender between the two RBP regulatory subtypes, indicating that individuals of different genders might have different RBP regulatory patterns, corresponding to different periodontal immune phenotypes, and possibly associated with distinct genetic susceptibility to periodontitis. More information on clinical phenotype and prognosis are expected to draw further conclusions.

## 5. Conclusion

Our study depicted the correlations among RBPs and immune microenvironment and biological reactions in periodontitis, with strong correlations of the RBPs ISG20 and ESRP1 with activated B cell infiltration, BCR signaling activation, and HLA-DOB expression. These findings indicated that RBP-mediated regulation of immune microenvironment as an important mechanism in the pathogenesis of periodontitis, which might inspire development of new therapeutic approaches.

## Data Availability

The data that support the findings of this study are available in https://www.ncbi.nlm.nih.gov/geo/query/acc.cgi?acc=gse16134.

## Supplementary Materials

Supplementary Materials Supplementary figures (FigureS1-S3) were uploaded entitled “supplementary figures.docx.” Supplementary tables (Table S1-S12) were uploaded entitled “Table S1: significantly dysregulated RBPs .txt.” “Table S2: results of univariate logistic regression.txt.” “Table S3: results of multivariate logistic regression.txt.” “Table S4: diversity of immunocytes between healthy and periodontitis samples.txt.” “Table S5: correlation analysis between immnuocytes and RBPs.txt.” “Table S6: diversity of immune pathways between healthy and periodontitis samples.txt.” “Table S7: Correlations between RBPs and immune pathways.txt.” “Table S8: diversity of HLA expression between healthy and periodontitis samples.txt.” “Table S9: correlation between RBPs and HLA expression.txt.” “Table S10: RBP regulatory subtypes.txt.” “Table S11: RBP regulatory subtype related genes.txt.” “Table S12: gene significance and module membership.txt,” respectively, with their filename indicating the contents of the tables.
