# Supplementary material for: Crosstalk between RNA-Binding Proteins and Immune Microenvironment Revealed Two RBP Regulatory Patterns with Distinct Immunophenotypes in Periodontitis
Source: J Immunol Res. 2021 Jul 5;2021:5588429. doi: 10.1155/2021/5588429 (PMC8275429; doi:10.1155/2021/5588429)
Supplement: Supplementary Materials — Supplementary figures (FigureS1-S3) were uploaded entitled “supplementary figures.docx.” Supplementary tables (Table S1-S12) were uploaded entitled “Table S1: significantly dysregulated RBPs .txt.” “Table S2: results of univariate logistic regression.txt.” “Table S3: results of multivariate logistic regression.txt.” “Table S4: diversity of immunocytes between healthy and periodontitis samples.txt.” “Table S5: correlation analysis between immnuocytes and RBPs.txt.” “Table S6: diversity of immune pathways between healthy and periodontitis samples.txt.” “Table S7: Correlations between RBPs and immune pathways.txt.” “Table S8: diversity of HLA expression between healthy and periodontitis samples.txt.” “Table S9: correlation between RBPs and HLA expression.txt.” “Table S10: RBP regulatory subtypes.txt.” “Table S11: RBP regulatory subtype related genes.txt.” “Table S12: gene significance and module membership.txt,” respectively, with their filename indicating the contents of the tables. [file 5588429.f1.zip › 5588429.f1/supplementary figures (1).docx]

**
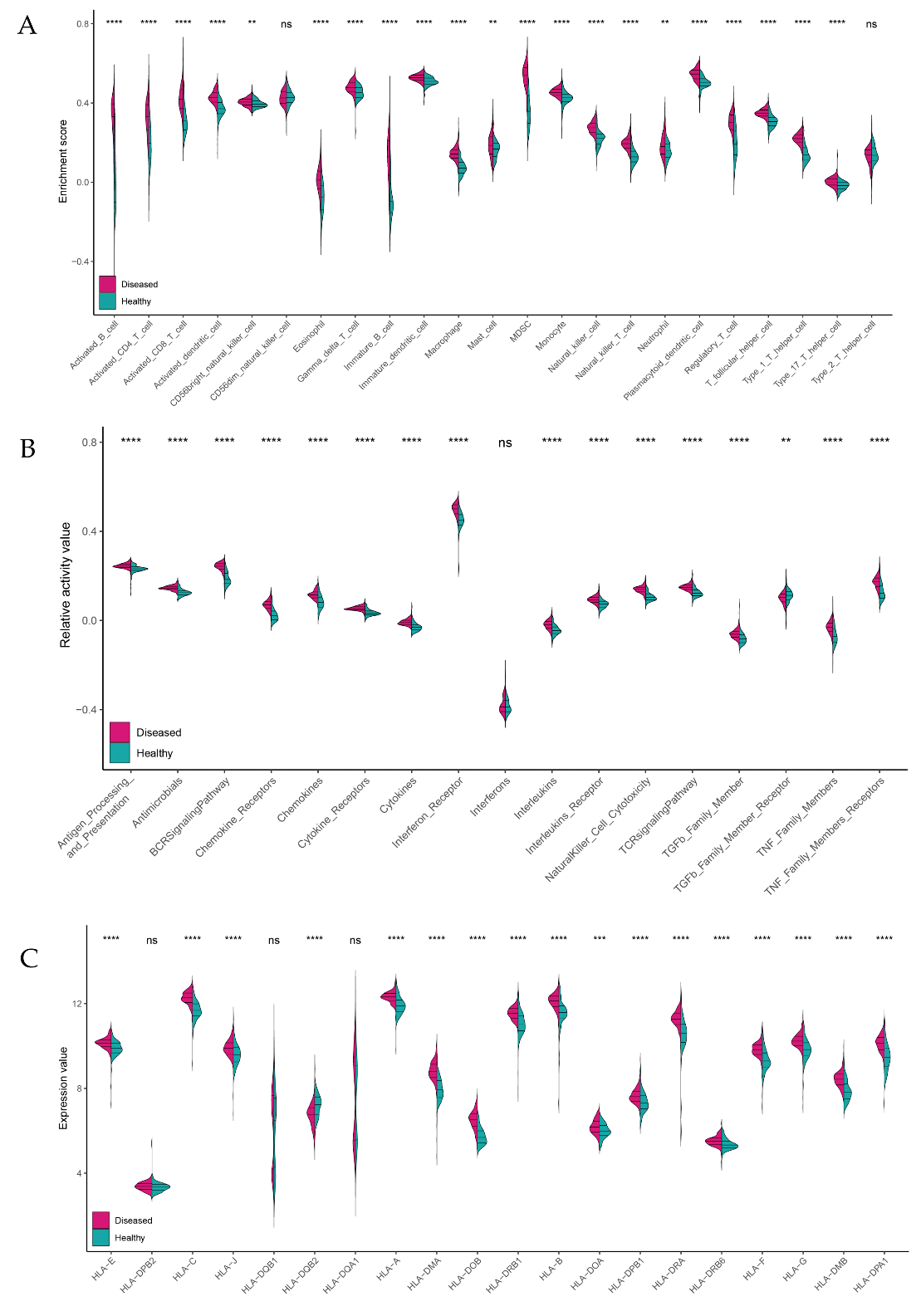
Figure S1. Immune microenvironment in periodontitis vs. healthy samples.** (A) The relative abundance of immunocytes in periodontitis and periodontally healthy samples. (B) The activity of immune-related pathways in periodontitis and periodontally healthy samples. (C)The expression levels of HLAs in periodontitis and periodontally healthy samples.


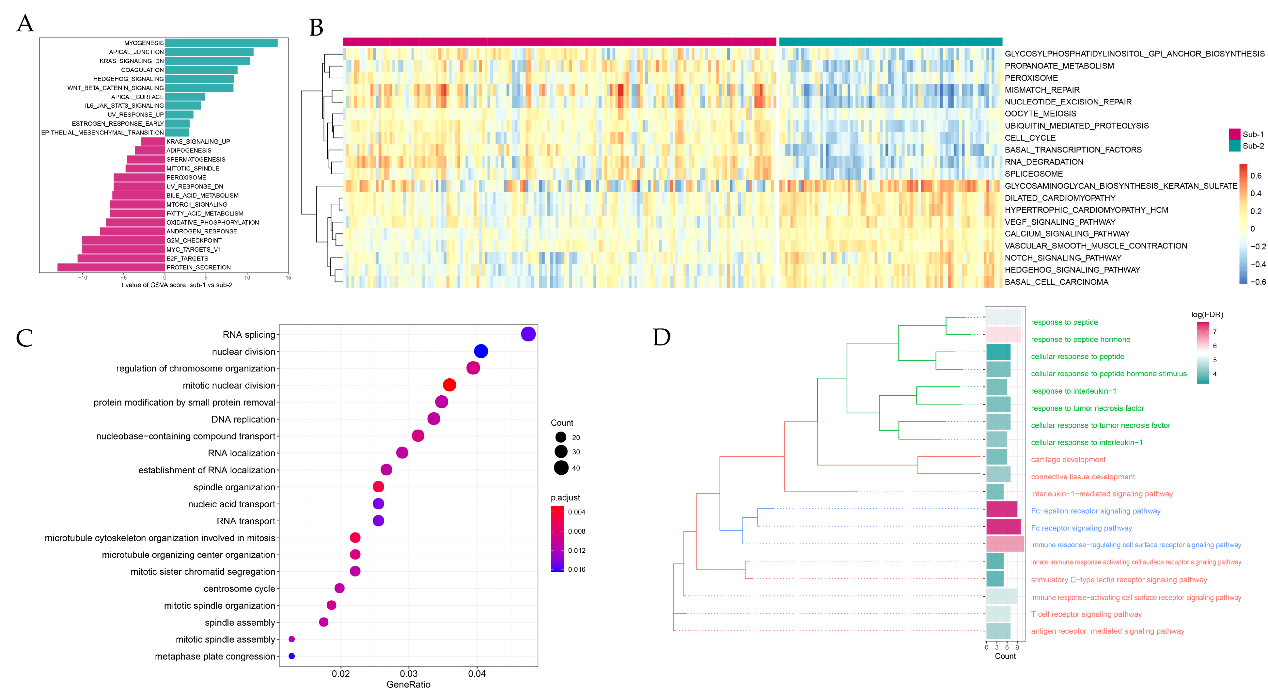
**Figure S2. Biological distinctions between the two RBP regulatory patterns.**  (A) Biological distinctions of the RBP regulatory patterns represented by HALLMARKS. (B) KEGG pathway scores for the two RBP regulatory patterns. (C) GO-BP functional enrichment analysis of the RBP-phenotype related genes between the two RBP regulatory patterns. (D) GO-BP functional enrichment analysis of the RBP-phenotype related immune genes. The terms were clustered according to the function.


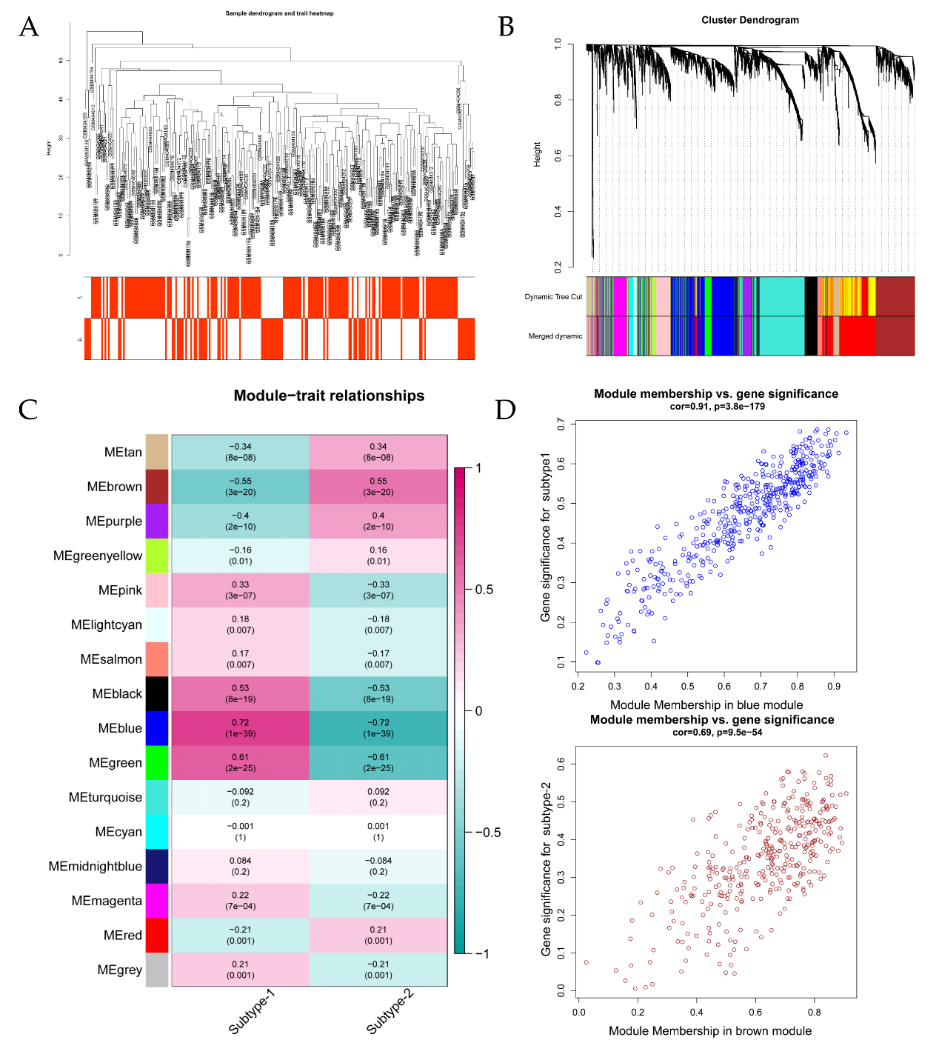


**Figure S3. Gene modules related to RBP regulatory patterns.** (A) The sample clustering was based on the expression data of periodontitis samples. The top 3000 of variation genes were used for the analysis by WGCNA and outlier samples were excluded. (B) Gene dendrogram obtained by average linkage hierarchical clustering. The color row underneath the dendrogram shows the module assignment determined by the Dynamic Tree Cut, in which 16 modules were identified. (C) Heatmap of the correlation between module eigengenes and the RBP regulatory patterns. (D) Scatter plots of gene significance versus module membership in the two RBP regulatory patterns. Genes in the blue module had a high correlation with RBP regulatory subtype-1 and genes in the brown module had a high correlation with RBP regulatory subtype-2.
